# Improved outcome in children compared to adolescents and young adults after allogeneic hematopoietic stem cell transplant for acute myeloid leukemia: a retrospective study from the Francophone Society of Bone Marrow Transplantation and Cell Therapy (SFGM-TC)

**DOI:** 10.1007/s00432-021-03761-w

**Published:** 2021-09-04

**Authors:** Cécile Pochon, Marie Detrait, Jean-Hugues Dalle, Gérard Michel, Nathalie Dhédin, Yves Chalandon, Eolia Brissot, Edouard Forcade, Anne Sirvent, Faezeh Izzadifar-Legrand, Mauricette Michallet, Cécile Renard, Ibrahim Yakoub-Agha, Fanny Gonzales, Jacques-Olivier Bay, Justyna Kanold, Jérome Cornillon, Claude Eric Bulabois, Marie Angoso, Stéphanie Nguyen, Marie Balza, Patrice Chevallier, Fanny Rialland, Ali Bazarbachi, Yves Beguin, Anne Huynh, Anne-Lise Ménard, Pascale Schneider, Bénédicte Neven, Catherine Paillard, Nicole Raus, Eliane Albuisson, Thomas Remen, Marie-Thérèse Rubio

**Affiliations:** 1 CHRU de Nancy, hôpitaux de Brabois, service d’oncohématologie pédiatrique, 54500 Vandœuvre-lès-Nancy, France; 2 CHRU de Nancy, hôpitaux de Brabois, service d’hématologie, 54500 Vandœuvre-lès-Nancy, France; 3 Biopôle de l’université de Lorraine, UMR 7365 CNRS-UL, IMoPa, 54500 Vandœuvre-lès-Nancy, France; 4 Hôpital Robert-Debré, Université Paris, département d’hémato-immunologie pédiatrique7-Paris Diderot, 5, rue Thomas-Mann, 75013 Paris, France; 5 Pediatric Hematology Department, Hopital de La Timone, Marseille, France; 6 Unité d’Hématologie-Adolescents et jeunes adultes, Hôpital Saint-Louis, EA-3518, Université Paris, 7-Denis Diderot, Paris, France; 7 Service d’Hématologie, Hôpitaux Universitaires de Genève, Université de Genève, 4, rue Gabrielle-Perret-Gentil, 1211 Genève and faculté de médecine, Geneva, Switzerland; 8 Service d’Hematologie Clinique, Saint-Antoine Hospital, AP-HP, Sorbonne University, and INSERM UMRs 938, Paris, France; 9 CHU Bordeaux, service d’hematologie et therapie Cellulaire, 33000 Bordeaux, France; 10 Hôpital Arnaud-de-Villeneuve, service d’onco-hématologie pédiatrique, 371, avenue du Doyen-Gaston-Giraud, 34090 Montpellier, France; 11 Institut Paoli-Calmette, unité de greffe, 232, boulevard de Sainte-Marguerite, 13009 Marseille, France; 12 Hematology Department, Centre Léon Bérard, Lyon, France; 13 Institute of Hematology and Oncology Paediatrics, Hospices Civils de Lyon, Lyon, France; 14 CHRU de Lille, unité d’allogreffe de CSH, maladies du sang, 59037 Lille, France; 15 Université de Lille 2, Inserm U995, LIRIC, 59000 Lille, France; 16 CHU de Lille, hématologie pédiatrique, 59000 Lille, France; 17 Department of Hematology, CHU de Clermont Ferrand, Clermont Ferrand, France; 18 Department of Pediatric Oncology and Hematology, Hôpital Estaing, Clermont-Ferrand, France; 19 Institut de Cancérologie Lucien-Neuwirth, département d’hématologie clinique, 108 Bis, avenue Albert-Raimond, 42271 St-Priest-en-Jarez, France; 20 CHU Grenoble, Grenoble, France; 21 Hôpital d’enfants, unité d’hématologie oncologie pédiatrique, place Amélie-Raba-Léon, 33000 Bordeaux, France; 22 Sorbonne Université, Groupe Hospitalier Pitié-Salpêtrière, centre d’immunologie et des maladies infectieuses (CIMI-Paris), service d’hématologie clinique, UPMC CR7, CNRS ERL8255, Inserm U1135, 75013 Paris, France; 23 Hematology Department, HCL, Hôpitaux Lyon-Sud, Pierre-Bénite, France; 24 CHU Nantes, Nantes, France; 25 Pediatric Hematology Department, CHU de Nantes, Nantes, France; 26 Department of Internal Medicine, American University of Beirut, Beyrouth, Lebanon; 27 Department of Haematology, CHU and University of Liège, Liège, Belgium; 28 Institut Universitaire du Cancer, Toulouse, France; 29 Centre Henri-Becquerel, département d’hématologie clinique, rue d’Amiens, 76038 Rouen, France; 30 Service d’hémato-oncologie pédiatrie, Hôpital Charles-Nicolle, CHU, 1, rue Germont, 76031 Rouen cedex, France; 31 Service d’immuno-Hématologie Pédiatrique, Hôpital Necker-Enfants-Malades, 149-161, rue de Sèvres, 75743 Paris Cedex 15, France; 32 Department of Haematology, Hôpital de Haute-Pierre, 67200 Strasbourg, France; 33 Data Management of SFGMT-TC, HCL, Hôpitaux Lyon Sud, Pierre Bénite, France; 34 CHRU-Nancy, DRCI, Département MPI, Unité de Méthodologie, Data Management et Statistique UMDS, 54000 Nancy, France

**Keywords:** Acute myeloblastic leukemia, Allogeneic hematopoietic stem cell transplantation, Children, Adolescent and post-adolescent patients, Young adults, Outcome, Acute GVHD, Chronic GVHD

## Abstract

**Background:**

There are currently few data on the outcome of acute myeloid leukemia (AML) in adolescents after allogeneic HSCT. The aim of this study is to describe the outcome and its specific risk factors for children, adolescents and young adults after a first allogeneic HSCT for AML.

**Methods:**

In this retrospective study, we compared the outcome of AML patients receiving a first allogeneic HSCT between 2005 and 2017 according to their age at transplantation’s time: children (< 15 years, *n* = 564), adolescent and post-adolescent (APA) patients (15–25 years, *n* = 647) and young adults (26–40 years; *n* = 1434).

**Results:**

With a median follow-up of 4.37 years (min–max 0.18–14.73 years), the probability of 2-year overall survival (OS) was 71.4% in children, 61.1% in APA patients and 62.9% in young adults (*p* = 0.0009 for intergroup difference). Both relapse and non-relapse mortality (NRM) Cumulative Incidence (CI) estimated at 2 years were different between the age groups (30.8% for children, 35.2% for APA patients and 29.4% for young adults—*p* = 0.0254, and 7.0% for children, 10.6% for APA patients and 14.2% for young adults, p < 0.0001; respectively). Whilst there was no difference between the three groups for grade I to IV acute GVHD CI at 3 months, the chronic GVHD CI at 2 years was higher in APA patients and young adults (31.4% and 36.4%, respectively) in comparison to the children (17.5%) (*p* < 0.0001). In multivariable analysis, factors associated with death were AML cytogenetics (HR1.73 [1.29–2.32] for intermediate risk 1, HR 1.50 [1.13–2.01] for intermediate risk 2, HR 2.22 [1.70–2.89] for high cytogenetics risk compared to low risk), use of TBI ≥ 8 Grays (HR 1.33 [1.09–1.61]), disease status at transplant (HR 1.40 [1.10–1.78] for second Complete Remission (CR), HR 2.26 [1.02–4.98] for third CR and HR 3.07 [2.44–3.85] for active disease, compared to first CR), graft source (HR 1.26 [1.05–1.50] for Peripheral Blood Stem Cells compared to Bone Marrow) and donor age (HR 1.01 (1–1.02] by increase of 1 year).

**Conclusion:**

Age is an independent risk factor for NRM and extensive chronic GVHD. This study suggests that APA patients with AML could be beneficially treated with a chemotherapy-based MAC regimen and bone marrow as a stem cells source.

**Supplementary Information:**

The online version contains supplementary material available at 10.1007/s00432-021-03761-w.

## Background

There are currently few available data on the outcome of Adolescent and Post-Adolescent (APA) patients after allogeneic hematopoietic stem cell transplantation (HSCT) for acute myeloid leukemia (AML) (Jaime-Pérez et al. 2018; Tomizawa et al. 2017; Canner et al. 2013; Nasir et al. 2017; Pemmaraju et al. 2016).

Acute myeloid leukemia represents about 15–20% of childhood leukemia, approximately 33% of adolescent leukemia, and approximately 50% of adult leukemia. After a peak in the first 2 years of life, the annual incidence of AML increases slowly and gradually after the age of 9 years old (Appelbaum et al. 2006). Pediatric and adult AML patients overall share biological parameters although some differences have not been systematically reviewed to date. Acute myeloid leukemia treatment has considerably improved for all age’s groups over the last 20 years, particularly through the improvement of allogeneic HSCT techniques. However, outcome appears to worsen with increased patient age. In comparison with pediatric and adult groups, the data of allogeneic HSCT for AML in adolescents are rare since they usually represent a small percentage within the cohorts of adults or children. However, these data are important since APA patients are treated in both pediatric and adult hematology departments, using different conditioning regimens—either myeloablative conditioning (MAC) or reduced-intensity conditioning (RIC)—and different graft sources, which might influence the disease outcome. We conducted a large retrospective study based on the French speaking Society for Bone Marrow Transplantation and Cell Therapy (SFGM-TC) registry to analyze and compare the outcome of AML patients classified in three age groups: children (0–14 years), APA patients (15–25 years) and young adults up to 40 years (26–40 years), who received an allogeneic HSCT from January 2005 to December 2017. In addition, we determined the factors influencing Overall Survival (OS), Event-Free Survival (EFS), Non-Relapse Mortality (NRM), Graft *Versus* Host Disease (GVHD) and Relapse Free Survival (GRFS) in the three age groups.

## Methods

This is a retrospective multicenter analysis using the data set from the Francophone Society of Bone Marrow Transplantation and Cellular Therapy (SFGM-TC) registry. The study protocol was approved by the scientific council of the SFGM-TC and complied with French regulatory requirements. The study was conducted according to the Declaration of Helsinki. All patients provided written informed consent authorizing the use of their personal information for research purposes.

We collected data from all patients up to the age of 40 years old included in the SFGM-TC registry from January 2005 to December 2017 who received a first allogeneic HSCT for treatment of AML.

Inclusion criteria were: patients younger than 41 years old who accepted to be registered in the SFGM-TC registry, treated with a first allogeneic HSCT for AML in first or further remission and also in refractory state. We included patients during the period from 2005 to 2017. The hematopoietic stem cell source was indifferently peripheral blood or bone marrow or cord blood. Forty-three centers accepted to participate in this study.

Risk staging of AML was reported according to the 2016 European Leukemia Net classification: low-risk was defined as CBF leukemia: t(8; 21)(q22; q22) RUNX1-RUNX1T1 or inv(16)(p13.1q22) CBFB-MYH11, or leukemia with biallelic mutations of CEBPA, or leukemia with normal karyotype and mutated NPM1 without FLT3-ITD. Intermediate risk 1 was defined as leukemia with normal karyotype with either mutated NPM1 and FLT3-ITD (mutant/wild-type mutation ratio > 0.3), or wild-type NPM1 and mutated FLT3-ITD, or wild-type NPM1 without FLT3-ITD. Intermediate risk 2 was defined as t(9; 11)(p21.3; q23.3) MLLT3-KMT2A or cytogenetic abnormalities not classified as favorable or adverse. High-risk group was defined as t(6; 9)(p23; q34.1) DEK-NUP214, t(v; 11q23) KMT2A rearranged, t(9; 22)(q34.1; q11.2) BCR-ABL1, inv(3)(q21.3; q26.2) or t(3; 3)(q21.3; q26.2) GATA2, MECOM (EVI1), complex karyotype (≥ 3), -5 or del (5q), − 7; − 17/abn(17p), mutated RUNX1, mutated ASXL1 (if not in low risk cytogenetics) and mutated TP53 (Stölzel et al. 2016). The conditioning regimen was considered Myeloablative Conditioning (MAC) if the total IV busulfan dose exceeded 12 mg/kg or the total fractionated body irradiation (TBI) dose exceeded 8 Grays. The combination of fludarabine 150 mg/m^2^ and IV busulfan 12.8 mg/kg (FB4) was defined as a reduced-toxicity MAC regimen. The other combinations have been defined as a Reduced-Intensity Regimen (RIC) (Bacigalupo et al. 2009).

Grading of acute GVHD was performed using the Glucksberg’s score (Glucksberg et al. 1974). Chronic GVHD was classified as limited or extensive according to previous published criteria (Filipovich et al. 2005).

### Statistical analysis

For variable description, categorical variables were expressed as numbers and percentages and discrete/continuous variables by median and extremes (min–max) values.

Comparisons by age groups (i.e., children, APA patients and young adults) were performed using Chi-Squared or Fisher exact tests as appropriate for categorical variables and Kruskal–Wallis test for discrete/continuous variables.

Inter-groups comparisons for the following time-dependent variables were performed using Kaplan–Meier analyses: Overall Survival (OS), event-free survival (EFS) and GRFS.

Inter-groups comparisons for the following time-dependent variables were performed using Gray’s test for equality of Cumulative Incidence Functions (CIF): Non-Relapse Mortality (NRM) (Competitive Risk (CR) = relapse), acute GVHD (CR = death), grade II–IV acute GVHD (CR = death), grade III to IV acute GVHD (CR = death), chronic GVHD (CR = death) and extensive chronic GVHD (CR = death).

For the mentioned time-dependent events, probability of OS, EFS and GRFS at 2 years were computed and intergroup survival distributions were compared using log-rank tests. Cumulative incidences at the following time points (event) were estimated after considering death or relapse occurrence as competitive events: 3 months (acute GVHD), 1 year (chronic GVHD), 2 years (relapse, death not related to relapse and GRFS), and compared using Gray’s test.

For each time-dependent variable, independent risk factors were explored using multivariable Cox regression model after censoring the follow-up at time of competing event when appropriate. The following variables were considered as potential predictors for all models: age, pediatric or adult center, recipient’s gender, recipient’s CMV status, cytogenetics, extramedullary disease, previous autologous transplantation, delay between AML diagnosis and HSCT, myeloablative, reduced-intensity or sequential conditioning regimen, use of antithymoglobulins (ATG), total body irradiation (< 8 grays or ≥ 8 grays), use of methotrexate or mofetil mycophenolate, use of high dose cyclophosphamide after transplantation, disease status at transplantation, graft source, HLA matching, donor’s gender, age and CMV serology, acute GVHD occurrence and staging, chronic GVHD occurrence and staging, disease status at the time of last news, delay from HSCT to relapse, Donor Lymphocyte Infusions (DLI).

Proportional hazard assumption was checked for each time-to-event outcome—predictor combination, and if violated, a time-dependant interaction term was added in the model. Then, univariate analyses were conducted including the interaction term and after censoring the follow-up to the competitive event when required. Independent risk-factors of each time-to-event outcome were explored using multivariable Cox regression model with significance levels for entry (SLE) and for stay (SLS) of 0.20 and 0.05, respectively.

Statistical analyses were performed using SAS version 9.4. The level of statistical significance was set at 0.05.

## Results

### Transplantation characteristics and comparison of patients in the three age groups

We analyzed data from 2645 patients aged from 0 to 40 years old, who received a first allogeneic HSCT between January 2005 and December 2017 from 43 SFGM-TC centers. The patient’s characteristics are presented in Table 1.

**Table 1:** Patient and transplantation characteristics

*N* = 2645	Group 1 (0–14 years) *N* = 564 (%)	Group 2 (15–25 years) *N* = 647 (%)	Group 3 (26–40 years) *N* = 1434 (%)	*P*
Patients characteristics				
Male	315 (56.0%)	346 (53.5%)	716 (50.0%)	0.0431
Female	249 (44.0%)	301 (46.5%)	717 (50.0%)	
Median (min-max) age at AML diagnosis (years)	6.7 (0.0–14.89)	20.0 (0.6–25.6)	33.8 (13.6–40.0)	< 0.0001
Median (min-max) age at transplantation (years)	7.6 (0.3–15)	20.9 (15.0–25.9)	34.6 (26.0–40.0)	< 0.0001
Cytogenetics (*n* = 1972)				< 0.0001
Low risk	76 (19.0%)	99 (20.5%)	234 (21.5%)	
Intermediate risk 1	45 (11.0%)	100 (20.5%)	283 (26.0%)	
Intermediate risk 2	99 (24.5%)	124 (25.5%)	243 (22.5%)	
High risk	183 (45.5%)	163 (33.5%)	323 (30.0%)	
Extra-medullary involvement at diagnosis	288 (51.0%)	224 (35.0%)	463 (32.0%)	< 0.0001
Status at transplantation				< 0.0001
CR1	327 (60.0%)	396 (63.0%)	899 (64.5%)	
≥ CR2	170 (31.0%)	141 (22.5%)	259 (18.5%)	
Refractory	49 (9.0%)	91 (14.5%)	237 (17.0%)	
Type of donor (% among groups)				< 0.0001
MRD/Syngeneic	198 (35.2%)	233 (36.0%)	555 (38.8%)	
MUD	198 (35.2%)	224 (34.6%)	540 (37.7%)	
MMUD	149 (26.5%)	149 (23.0%)	250 (17.5%)	
Haploidentical	18 (3.2%)	41 (6.3%)	86 (6.0%)	
Donor age (median, min-max)	21.96 (0.07–57.94)	27.18 (0.08–64.16)	34.57 (1.54–72.96)	< 0.0001
Source of stem cells				< 0.0001
Bone marrow	356 (63.2%)	260 (40.2%)	404 (28.2%)	
Peripheral blood stem cell	55 (9.8%)	297 (45.9%)	894 (62.3%)	
Cord blood	152 (27%)	90 (13.9%)	136 (9.5%)	
Conditioning regimen				< 0.0001
MAC	522 (95.3%)	490 (79%)	1063 (76.1%)	
RIC	20 (3.6%)	76 (12.3%)	197 (14.1%)	
Sequential	6 (1.1%)	54 (8.7%)	137 (9.8%)	
TBI based ( ≥ 8Grays)	42 (7.5%)	166 (25.7%)	367 (25.6%)	< 0.0001
Description of MAC conditioning regimen				< 0.0001
BuCy	403 (73.0%)	221 (34.4%)	428 (30.1%)	
FB4	34 (6.1%)	93 (14.5%)	262 (18.3%)	
TBI-Cy	27 (4.9%)	133 (20.7%)	309 (21.7%)	
Other	88 (16.0%)	195 (30.4%)	424 (29.9%)	
GvHD prophylaxis				< 0.0001
CSA-MTX	167 (31.6%)	305 (51.0%)	729 (54.5%)	
CSA-MMF	72 (13.6%)	176 (29.4%)	406 (30.4%)	
CSA alone	290 (54.8%)	117 (19.6%)	202 (15.1%)	
ATG in vivo T depletion	237 (42.0%)	277 (42.8%)	702 (49.0%)	0.0036
Post-Transplant High-dose Cyclophosphamide (PTCy)	15 (2.6%)	40 (6.2%)	87 (6.1%)	0.0056
Use of PT-Cy in Haploidentical HSCT	9 (50%)	30 (73.2%)	54 (62.8%)	0.2137
Donor lymphocyte infusions	34 (6.0%)	61 (9.4%)	180 (12.6%)	< 0.0001
Indication of DLI				
Preemptive	12 (35.3%)	20 (32.8%)	59 (32.8%)	0.37
After relapse	21 (61.8%)	31 (50.8%)	102 (56.7%)	

*ATG * Anti-thymoglobulin, *BuCY * busulfan 12.8–19.2mg/kg and cyclophosphamide 120 or 200 mg/kg, *CR * Complete remission, *CSA* ciclosporine A, *FB4* fludarabine 120–160 mg/m^2^ and busulfan 12.8–19.2 mg/kg , *GvHD* Graft-versus-host Disease, *Haploidentical-HSCT * Haploidentical hematopoietic stem cell transplantation, *MAC* Myeloablative Conditioning, *MMF* mycophenolate mofetil, *MMUD * Mismatched Unrelated Donor (HLA < 10/10), *MRD * Matched Related Donor, *MTX* methotrexate, *MUD * Matched Unrelated Donor (HLA 10/10), *RIC* Reduced-Intensity Regimen, *TBI * Total Body Irradiation

The median follow-up of the study was 2.4 years (min–max 1 day–14.7 years), from the time of transplant to death or latest news date. Among alive patients, the median follow-up of the study was 4.37 years (min–max 0.18–14.73 years). Three age groups were assessed: 564 children aged from 0 to 14 years, 647 Adolescent and Post-Adolescent (APA) patients aged from 15 to 25 years, and 1434 young adult patients aged from 26 to 40 years.

The cytogenetics risk, the extramedullary involvement at diagnosis and the disease status at transplant were different in the three groups (*p* < 0.0001 for all analyses). The conditioning regimen was mainly myeloablative in the three groups (79% in APA patients, 76.1% in young adults and 95.3% in children), but APA patients and young adults received more often RIC regimen than the children (12.3 and 14.1% versus 3.6%). The use of TBI equal and over 8 Grays was different according to the age groups (*p* < 0.0001), APA patients and young adults received more TBI (25.7 and 25.6% respectively) than the children (7.5%). The use of Anti-Thymoglobulin (ATG) was different in the three groups (*p* = 0.0036): it was slightly less often used in APA patients and children compared to the young adults (42.8% and 42% versus 49%, respectively). Post-Transplant High Dose Cyclophosphamide (PT-Cy) was used in a minority of cases, 2.6% in children, 6.2% in APA patients and 6.1% in young adults (*p* = 0.0056 as intergroup comparison). PT-Cy was not only dedicated to haploidentical transplantations: 63.6% patients that received PT-Cy, underwent a haploidentical transplant and 62.7% haploidentical transplant were followed by PT-Cy. We observed some significant differences in stem cell source between the three groups (*p* < 0.0001). Bone marrow (BM) was the main source in children (63.2%, followed by cord blood in 27%, and peripheral blood stem cells (PBSC) in only 9.8%), PBSC were the major source of HSCT in young adults (62.3%, followed by bone marrow in 28.2%) while APA patients received bone marrow in 40.2%, PBSC in 45.9% and cord blood in 13.9%. The donor’s age was also different in the three age groups (*p* < 0.0001).

### Engraftment and Graft-*versus*-Host Disease (GVHD)

The neutrophils recovery time up to 0.5 G/L differed between the three age groups (*p* < 0.0001) with a median (min–max) value of 20 days (4–61) in children, 19 days (1–66) in APA patients, and 18 days (1–108) in young adults. The platelets recovery time up to 20 G/L also differed in the three age groups (*p* < 0.0001) with a median (min–max) value of 21 days (3–181) in children, 18 days (1–124) in APA patients and 16 days (1–152) in young adults (Table 2).

**Table 2:** Patient outcomes according to age group

	Group 1 (0-14 years) *n* = 564	Group 2 (15-25 years) *n* = 647	Group 3 (26-40 years) *n* = 1434	*P*
Median (min-max) follow-up among alive patients in years	4.30 (0.21–14.60)	4.49 (0.18–14.73)	4.37 (0.25–14.24)	0.7381
Engraftment				
Median (min-max) duration of PNN> 0.5G/L (days)	20 (4–61)	19 (1–66)	18 (1–108)	**<** **0.0001**
Median (min-max) duration of Platelets > 20G/L (days)	21 (3–181)	18 (1–124)	16 (1–152)	**<** **0.0001**
Probability at 2 years (%)				
OS [95% CI]	71.4 [67.4–75.0]	61.1 [57.1–64.8]	62.9 [60.3–65.4]	**0.0009**
EFS [95% CI]	61.5 [57.2–65.5]	53.7 [49.7–57.6]	55.8 [53.1–58.4]	**0.0186**
GRFS [95% CI]	47.0 [42.7–51.1]	40.1 [36.2–44.0]	40.9 [38.3–43.5]	0.1107
Cumulative incidence (%)				
Grade I–IV acute GvHD at 3 m [95% CI]	55.7 [51.3–59.8]	49.3 [45.2–53.2]	50.4 [47.7–53.0]	0.0534
Grade II–IV acute GvHD at 3 m [95% CI]	37.8 [33.6–42.0]	34.6 [30.8–38.4]	33.8 [31.3–36.3]	0.1940
Grade III–IV acute GvHD at 3m [95% CI]	13.8 [11.0–17.0]	13.1 [10.6–16.0]	12.2 [10.6–14.1]	0.6097
Chronic GvHD at 2 years [95% CI]	17.5 [14.4–20.8]	31.4 [18.4–27.8]	36.4 [33.9–38.9]	**<** **0.0001**
Extensive chronic GvHD at 2 years [95% CI]	7.1 [5.2–9.3]	12.5 [10.0–15.2]	15.4 [13.6–17.4]	**<** **0.0001**
Relapse at 2 years [95% CI]	30.8 [27.0–34.7]	35.2 [31.5–38.9]	29.4 [27.0–31.8]	**0.0254**
NRM at 2 years [95% CI]	7.0 [5.1–9.4]	10.6 [8.3–13.2]	14.2 [12.4–16.1]	**<** **0.0001**

Bold indicates statistical significance (*p* < 0.05)

*OS* overall survival, *EFS* event free survival, *GvHD* graft versus host disease, *NRM* non relapse mortality, *GRFS* graft *versus* host disease and relapse free survival

**Fig. 1:**
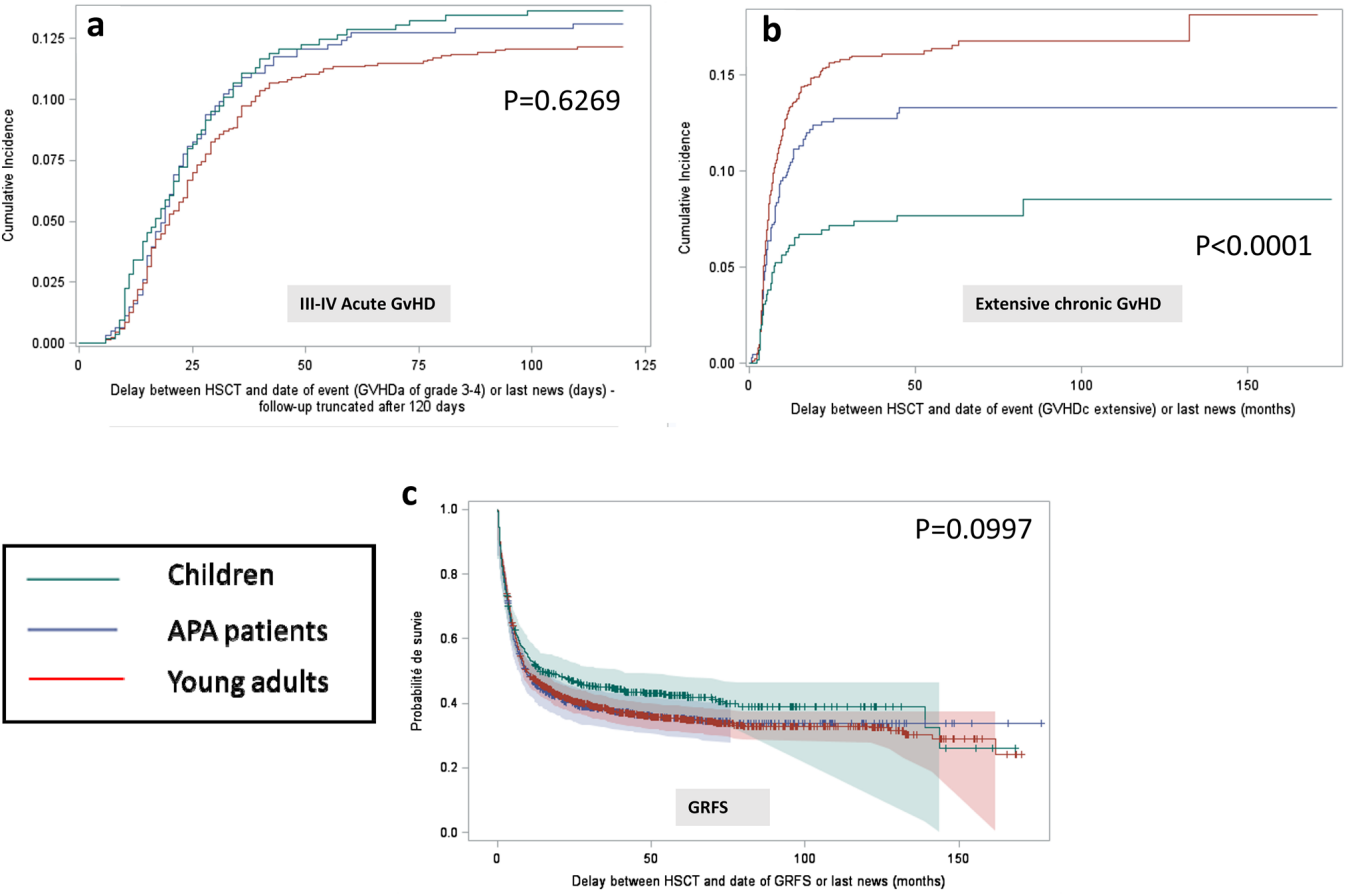
GVHD and GRFS **a** Cumulative incidence of acute GVHD for the 3 groups of age. **b** Cumulative incidence of extensive chronic GVHD for the 3 groups of age. **c** GVHD and Relapse Free Survival (Kaplan-Meïer curves) for the 3 groups of age

The grade I–IV acute GVHD cumulative incidence (CI) at 3 months was estimated to 55.7% for children, 49.3% for APA patients and 50.4% for young adults, the difference in the three age groups was close to being significant (*p* = 0.0534) (Table 2). Moreover, considering chronic GVHD, the CI at 2 years of follow-up showed a significant statistical difference in the three age groups (*p* < 0.0001), APA patients and young adults experiencing more chronic GVHD (31.4% and 36.4%, respectively) in comparison to the children (CI 17.5%) (Table 2, Fig. 1a, b).

The independent risk factors associated with grade II to IV acute GVHD were: the HLA matching (higher risk for mismatched unrelated donors followed by matched unrelated donors and haploidentical donors compared to the sibling donors), active disease at transplant time and the use of TBI ≥ 8 Grays. The protective factors were: the use of cord blood and PBSC, compared to the bone marrow, the use of ATG, the use of Post-Transplant High-Dose Cyclophosphamide (PT-Cy) and methotrexate in addition to cyclosporine in GVHD prophylaxis (Table 3).

**Table 3:** Multivariable analyses of risk factors for acute and extensive chronic GVHD occurrence

	aGvHD II–IV		aGvHD III–IV		Extensive cGvHD	
Variable	HR [95% IC]	*p*	HR [95% IC]	*p*	HR [95% IC]	*p*
Group of age	ns					**0.0131**
0–14 years					1	
15–24 years					1.66[1.05–2.61]	
25–40 years			ns		1.92[1.24–2.97]	
CMV Matching (D/R)	ns			**0.0078**	ns	
−/−				1		
+/−				0.77 [0.47–1.26]		
−/+				1.48 [1.03–2.12]		
+/+				1.51 [1.07–2.15]		
Disease status at transplantation						
CR 1	1	< **0.0001**	1	**< 0.0001**	ns	
CR 2	0.79 [0.63–0.99]		0.61[0.41–0.91]			
CR3	0.51 [0.12–2.06]		0.66[0.09–4.75]			
Active disease	1.59 [1.26–2.00]		1.91[1.35–2.70]			
HLA matching		**< 0.0001**		<**0.0001**	ns	
Matched sibling donor	1		1			
Haploidentical donor	1.48 [0.89–2.44]		1.43[0.75–2.71			
Matched unrelated donor	2.10 [1.69–2.61]		2.38[1.65–3.42]			
Mismatched unrelated donor	2.59 [1.96–3.41]		2.70[1.80–4.05]			
Donor age	ns					**0.0415**
Source of stem cells		< **0.0001**	ns			**0.0085**
Bone marrow	1				1	
PBSC	0.88 [0.72–1.06]				1.43[1.09–1.87]	
Cord blood	0.41 [0.29–0.58]					
Myeloablative TBI (≥ 8 Grays)	8	**0.0023**	ns		ns	
No	1					
Yes	1.36 [1.11–1.67]					
GvHD prophylaxis		**0.0017**	ns		ns	
CsA-alone	1					
CsA-MTX	0.70 [0.56–0.89]					
CsA-MMF	1.02 [0.79–1.30					
ATG		**< 0.0001**		**< 0.0001**		**< 0.0001**
No	1		1		1	
Yes	0.34 [0.24–0.47]		0.47[0.35–0.63]		0.55[0.42–0.71]	
HD cyclophosphamide post		**0.0370**	ns			**0.0051**
HSCT						
No	1				1	
Yes	0.60 [0.37–0.97]				0.31 [0.13–0.71]	

Bold indicates statistical significance (*p* < 0.05)

*ATG* antithymoglobulin, *BM* bone marrow, *CR* complete remission, *GvHD* graft-versus-host disease, *CsA* ciclosporine A, *MTX* methotrexate, *MMF* mycophenolate mofetil, *PBSC* peripheral blood stem cells, *TBI* total body irradiation *ns* variables not retained in the final model due to non-significance

Furthermore, independent risk factors associated with severe (grade III to IV) acute GVHD were: active disease at transplant time, HLA matching (higher risk in case of mismatched unrelated donors followed by matched unrelated donors and haploidentical donors compared to the sibling donors), and recipient CMV seropositivity. The use of ATG decreased the risk of severe acute GVHD (Table 3).

An independent risk factor of chronic GVHD was identified: the age group, adults being at highest risk, and then APA patients, compared to the children. The use of PT-Cy decreased the risk of chronic GVHD. As far as extensive chronic GVHD is concerned, the graft source (PBSC compared to bone marrow) and the increasing of donor’s age were also an independent risk factor in addition to the age group (young adults and APA patients, compared to the children). While the use of ATG or PT-Cy was an independent protective factor in extensive chronic GVHD (Table 3).

### Relapse

With a median follow-up of 2.4 years (min–max: 1 day–14.7 years), AML relapse occurred after HSCT in 193 children (35.2%), 247 APA patients (39.1%) and 474 young adults (34.5%). Median (min–max) delay from HSCT to relapse was 165.5 (1–4377) days in children, 151 days (7–2457) in APA patients and 182 days (1–4305) in young adults.

**Fig. 2:**
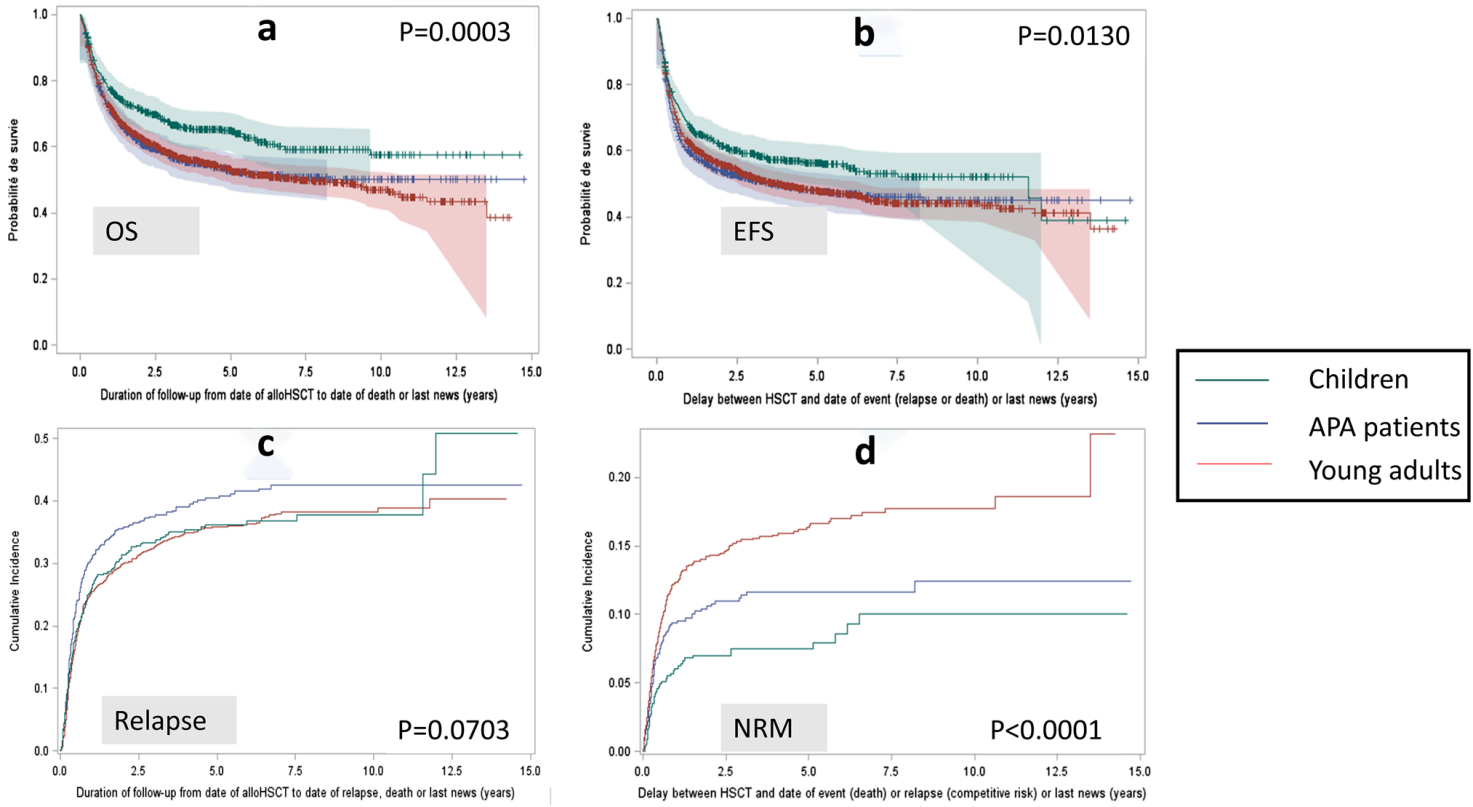
OS, EFS, Relapse and NRM **a** Overall Survival (Kaplan-Meïer curves) for the 3 groups of age. **b** Event Free Survival (Kaplan-Meïer curves) for the 3 groups of age. **c** Cumulative incidence of relapse for the 3 groups of age. **d** Cumulative incidence of Non Relapse Mortality for the 3 groups of age

The CI of relapse at 2 years differed in the three age groups (30.8% in children, 35.2% in APA patients and 29.4% in young adults—*p* = 0.0254) (Table 2, Fig. 2c).

The independent risk factors for relapse were: high cytogenetics risk, followed by intermediate risk 2 and 1 (compared to low cytogenetics risk), longer delay between diagnosis and HSCT, reduced-intensity conditioning regimens (compared to myeloablative conditioning regimens), active disease at transplant time, followed by third and second complete remission before HSCT (compared to the patients in first CR) (Table 4).

**Table 4:** Multivariable analyses of risk factors of death (OS model), relapse, non-relapse mortality (NRM) and GRFS

	OS^a^		Relapse		NRM^a^		GRFS^a^	
	HR [95%IC]	*p*	HR [95%IC]	*p*	HR [95%IC]	*p*	HR [95%IC]	*p*
Group of age						**0.0343**		
0–14 years					1			
15–24 years	NS		NS		1.32 [0.70–2.46]		NS	
25–40 years					1.88 [1.07–3.30]			
Sex Matching (D/R)								**0.0377**
M–M							1	
F–M							1.07 [0.88–1.30]	
M–F	NS		NS		NS		0.92 [0.76–1.10]	
F–F							1.22 [1.01–1.49]	
CMV Matching (D/R)								
−/−							1	**0.0130**
+/−	NS		NS		NS		0.84 [0.67–1.06]	
−/+							1.16 [0.96–1.41]	
+/+							1.18 [0.99–1.40]	
Cytogenetics		**< 0.0001**		**< 0.0001**		**0.0394**		**0.0184**
Low risk	1		1		1		1	
Intermediate 1	1.73 [1.29–2.32]		1.46 [1.10–1.93]		1.65 [1.00–2.72]		1.29 [1.02–1.63]	
Intermediate 2	1.50 [1.13–2.01]		1.58 [1.21–2.06]		1.05 [0.61–1.79]		1.15 [0.91–1.45]	
High risk	2.22 [1.70–2.89		1.87 [1.45–2.41]		1.69 [1.05–2.72]		1.37 [1.11–1.70]	
Delay between AML diagnosis and HSCT (days)	NS		1 [0.99–1.00]	**0.0192**	NS		**NS**	
Disease status at transplantation		**<0.0001**		**<0.0001**		**0.0004**		**< 0.0001**
CR 1	1		1		1		1	**< 0.0001**
CR 2	1.40 [1.10–1.78]		1.44 [1.10–1.88]		1.45 [0.95–2.22]		1.11 [0.89–1.38]	
CR 3	2.26 [1.02–4.98]		1.57 [0.62–3.99]		3.54 [1.25–9.95]		2.36 [1.16–4.76]	
Active disease	3.07 [2.44–3.85		2.76 [2.08–3.67]		2.27 [1.47–3.49]		**3**.04 [2.45–3.78]	
HLA matching						**0.0083**		**0.0032**
Matched sibling donor					1		1	
Haploidentical	NS		NS		2.55 [1.50–4.30]		1.41 [1.05–1.89]	
Matched unrelated donor					1.55 [1.06–2.27]		1.46 [1.18–1.81]	
Mismatched unrelated donor					1.45 [0.87–2.43		1.73 [1.29–2.34]	
Donor age	1.01 [1.00–1.02]	**0.0013**	NS		1.03 [1.01–1.04]	**0.0002**	1.01 [1.00–1.02]	**0.0049**
Source of stem cells		**0.0104**						**0.0465**
Bone marrow	1						1	
PBSC	1.26 [1.05–1.50]		NS		NS		1.16 [1.01–1.35]	
Conditioning regimen				**0.0179**	NS		NS	
Myeloablative			1					
Reduced–intensity			1.37 [1.07–1.75]					
Sequential			0.94 [0.65–1.37]					
Myeloablative TBI (≥ 8 Grays)		**0.0036**	NS		NS		NS	
No	1							
Yes	1.33 [1.09–1.61]							

Bold indicates statistical significance (*p* < 0.05)

*ATG* antithymoglobulin, *BM* bone marrow, *CR* complete remission, *GVHD* graft-versus-host disease, *MAC* myeloablative conditioning, methotrexate, *PBSC* peripheral blood stem cells, *TBI* total body irradiation *NS* variables not retained in the final model due to non-significance ^a^Factors are expressed as risk of mortality

Donor lymphocyte infusions (DLI) were rarely used in this cohort, either in prophylaxis or as curative treatment. Thirty-four (6%) children, 61 (9.4%) APA patients and 180 (12.6%) young adults received at least one DLI.

### Non-relapse mortality

The non-relapse mortality CI at 2 years was 7.0% in children, 10.6% in APA patients and 14.2% in young adults (*p* < 0.0001, Table 2, Fig. 2d) and the median (min–max) delay from HSCT to NRM was 0.34 (0.06–6.54) years, 0.33 (0.01–8.20) years and 0.45 (0–13.49) years, respectively.

The independent risk factors for NRM were: the age group (young adults followed by APA patients had a higher risk of NRM, compared to the children), the cytogenetics risk (high risk followed by intermediate risk 1and 2, compared to low risk), the disease status at transplant (third CR followed by active disease and second CR, compared to first CR), the HLA mismatch (haploidentical donors followed by mismatched unrelated donors and then matched unrelated donors, compared to the identical sibling donors) and the increasing of donor’s age (Table 4).

The causes of death (other than relapse) are described in Supplementary Table 1. Children mostly died of infections (*n* = 21, 10.7%), GVHD (*n* = 20, 10.2%) and pulmonary toxicity (*n* = 9, 4.6%). Adolescent and post-adolescent patients like young adults mostly died of infections (n = 53, 18.3% and n = 142, 21.9%; respectively), GVHD (*n* = 40, 13.8% and *n* = 125, 19.3%) and sinusoidal obstruction syndrome (*n* = 14, 4.8% and *n* = 21, 3.2%).

### OS and EFS

In this cohort, 1513 patients were alive (57.2%) after a median follow-up of 4.37 years, 368 children (65.2%), 358 APA patients (55.3%) and 787 young adults (54.9%). The OS was significantly different between the three groups (*p* = 0.0003, Fig. 2a). At 2 years, the probability of OS was 71.4% in children, 61.1% in APA patients and 62.9% in young adults (*p* = 0.0009 as intergroup difference, Table 2). In the subgroup of patients who did not relapse (n = 1641 patients), the probability of 2-year OS also differed in the three age groups (*p* < 0.0001) with 89.2% in children, 82.5% in APA patients and 78.2% in young adults.

The independent risk factors for death were: high cytogenetics risk, followed by intermediate risk 1 and 2 (compared to low risk), the use of TBI ≥ 8 Grays, active disease at transplant time followed by the patients in 3rd CR and 2nd CR (compared to the patients in 1st CR), the use of PBSC (compared to bone marrow), and the increase of donor’s age (Table 4).

The EFS was also different in the three age groups (*p* = 0.013, Fig. 2b) at 2 years with a rate of 61.5% in children, 53.7% in APA patients and 55.8% in young adults, *p* = 0.0186 (Table 2).

The independent risk factors for death or relapse were: high cytogenetics risk followed by intermediate risk 1 and 2, TBI ≥ 8 Grays in the conditioning regimen, active disease at transplant time followed by 3rd CR and 2nd CR, and the increasing of donor’s age.

### GRFS (Fig. 1c)

The GRFS, who was defined as survival without neither grade III-IV acute GVHD nor extensive chronic GVHD or relapse, was not significantly different in the three age groups (*p* = 0.0997, Fig. 1c). The probability of GRFS at 2 years was 47% in children, 40.1% in APA patients and 40.9% in young adults, *p* = 0.1107 (Table 2).

The independent protective factors for survival without neither disease nor GVHD were: CMV seronegative recipient (in particular the combination of positive donor and negative recipient), AML with low cytogenetics risk, male donor, transplant in 1st CR, bone marrow (compared to PBSC), younger and matched sibling donor (Table 4).

### Additional analysis

**Fig. 3:**
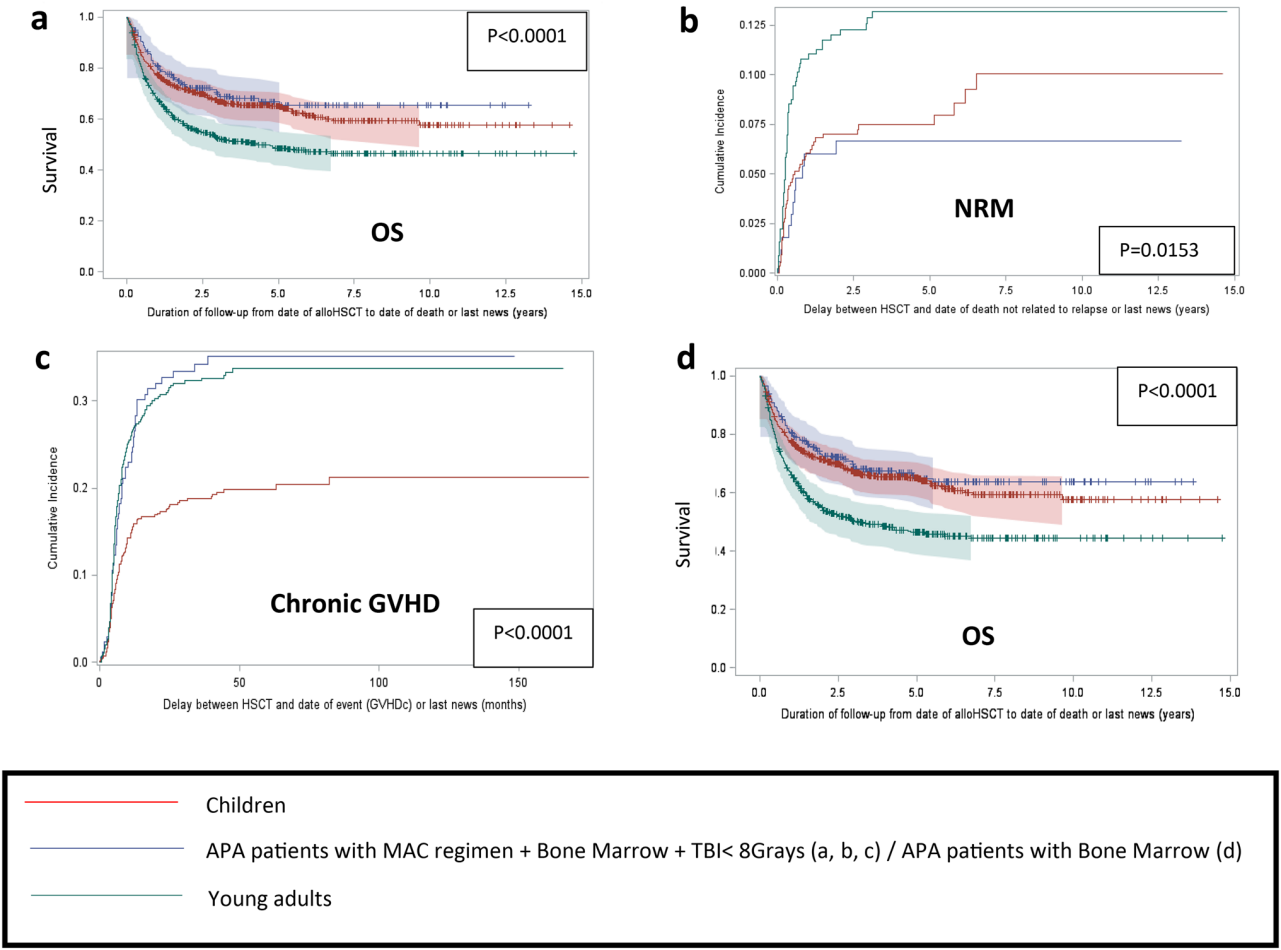
Better outcome of APA patients who received bone marrow grafts and a chemotherapy-based myeloablative conditioning regimen. **a** Overall Survival (Kaplan-Meïer curves) for APA patients who received a chemo-based MAC regimen and a bone marrow graft, compared to children and young adults. **b** Cumulative incidence of Non Relapse Mortality for APA patients who received a chemo-based MAC regimen and a bone marrow graft, compared to children and young adults. **c** Cumulative incidence of chronic GVHD for APA patients who received a chemo-based MAC regimen and a bone marrow graft, compared to children and young adults. **d** Overall survival (Kaplan-Meïer curves) for APA patients who received bone marrow grafts (whatever the conditioning regimen they received), compared to children and young adults

To describe more precisely the impact of the conditioning regimen and the stem cell source on OS, NRM and chronic GvHD for APA patients, we compared in a subgroup study the APA patients who received a chemotherapy-based MAC regimen and bone marrow as stem cell source, i.e., 171 patients (6.5%), other APA patients, i.e., 449 patients (17.2%) and the children, i.e., 564 patients (21.5%) (Fig. 3). We found a better survival for APA patients who received a chemotherapy-based regimen and bone marrow (*p* < 0.0001) (Fig. 3a), and the NRM was lower for this subgroup of patients (*p* = 0.0153) (Fig. 3b). However, the incidence of chronic GvHD was still lower for children (*p* < 0.0001) (Fig. 3c). Moreover, the OS was the same for children and APA patients who received bone marrow, compared to APA patients who received other stem cells sources (Fig. 3d).

## Discussion

This retrospective registry study from 2005 to 2017 showed that APA patients have a greater risk of NRM and chronic GVHD than children after allogeneic HSCT for AML. The relapse occurred slightly more frequently in the APA patient group, but age was not an independent factor for relapse. As far as NRM is concerned, we observed that the age group was an independent risk factor, but also the cytogenetics risk, the disease status, the HLA matching and the donor’s age; moreover, we observed a higher rate of chronic extensive GVHD in APA patients and young adults in comparison to children, with the age group as an independent risk factor.

Except for promyelocytic leukemia, APA patients with AML were described as presenting a poorer prognosis than children in a few studies due to a higher rate of relapse (Jaime-Pérez et al. 2018), higher risk of toxicity-related mortality (Tomizawa et al. 2017) owing to more frequent infections (Canner et al. 2013) and a higher early mortality rate (Nasir et al. 2017). Nevertheless APA patients had a better prognosis than adult AML patients (Pemmaraju et al. 2016). Age seems to be related to the outcome from childhood to adulthood (Appelbaum et al. 2006). Patients treated in pediatric trials had better outcomes than those treated on adult trials in an American study by the National Marrow Donor Program (NMDP), but age was a major confounding variable, making it harder to compare data sets by cooperative groups (Woods et al. 2013). Moreover, neither studies from the Nordic Society of Paediatric Haematology and Oncology (NOPHO), nor works from multi-center in Germany found no difference in outcomes for AML patients in overlapping age groups on pediatric versus adult protocols (Wennström et al. 2016; Büchner et al. 2009; Schlenk et al. 2003). Currently, there is a need for prospective studies to be able to issue a recommendation.

After allogeneic HSCT, a different survival rate for young AML patients was firstly reported from the International Bone Marrow Transplant Registry, from 1980 to 2005 (Majhail et al. 2012). Adolescent and post-adolescent patients were defined as aged 15 to 40 and had improved survival in comparison to older patients but also a worse prognosis compared to children of under 15 years of age. A further study from Minneapolis had reported no difference in children’s outcome compared to APA patients (aged 15–30) from 1995 to 2010, except for GVHD (Burke et al. 2014). A more recent study from the Japanese Group reported an inferior 5-year OS (54% versus 58%; *p* < 0.01) and an increased transplant-related mortality (TRM; 16% vs 13%, *p* = 0.02) in adolescent, post-adolescent and young adult patients (15–29 years) compared to children who received allogeneic HSCT for AML from 1990 to 2013 (Tomizawa et al. 2015). However, better HLA typing in recent years could eliminate this difference. Considering that last study, no difference in outcome of APA patients and children (OS, relapse-free survival and NRM) could be identified in the most recent period of their study between 2000 and 2013. This result is in contrast with our study on a more recent cohort of patients.

In our study, c*ytogenetics* risk was strongly related to OS, EFS, NRM and GRFS in multivariable analysis. However, children had more often high-risk cytogenetics, but did not experience a higher incidence of relapse, and had a higher EFS, which was in concordance with the study of Alloin et al. that found a significant survival improvement for children with unfavorable karyotype due to the decrease of relapse risk over time (Alloin et al. 2017). Furthermore, through age groups, there are observable differences in mutated genes, somatic structural variants and DNA methylation patterns (Bolouri et al. 2018). From the study of Boulouri et al., it is important to notice, for instance, that the prevalence of gene fusions and focal deletions in MBNL1 and ZEB2 is much higher in young patients than in adults and the mutations in DNMT3A and TP53 are highly uncommon in children compared to adult patients. In the future, all of these genetic observations should allow targeted and age-suited treatment of AML (Bolouri et al. 2018).

Disease status remains a strong independent factor in relapse, toxicity and death after HSCT. In all recent studies, more advanced disease is still correlated with death for both adults (Konuma et al. 2018; Gaballa et al. 2017) and children (Bitan et al. 2017) in spite of improvements in salvage therapies (Rasche et al. 2018); it is the same for the minimal residual disease (MRD) which is a strong prognostic factor before HSCT (Gilleece et al. 2021; Candoni et al. 2017). Refractory AML has a very bad prognosis despite efforts to develop new strategies such as sequential regimen, except in patients with low medullar blast burden in primary refractory AML (Steckel et al. 2018). In our study, the disease status at the time of transplant was correlated with OS, EFS, NRM and GRFS.

Young adults and APA patients received myeloablative TBI more frequently in their conditioning regimen whilst almost all children received myeloablative chemotherapy without any irradiation. TBI was an independent risk factor of overall mortality. These results are consistent with previous studies reporting that the use of TBI in the conditioning regimen of AML patients, in comparison to busulfan-based MAC regimen, was deleterious for adults and children, despite this being the contrary in ALL studies (Champlin 2013; Bredeson et al. 2013). This deleterious effect of TBI compared to chemotherapy with busulfan was mostly explained by a higher rate of NRM (Berranger et al. 2014) and chronic GVHD incidence (Copelan et al. 2013; Nagler et al. 2013).

The increased NRM for APA (and adult) patients in our study was also possibly a result of the higher cumulative incidence of chronic GVHD compared to children under 15 years old. As far as extensive chronic GVHD is concerned, it was independently associated with PBSC, that were used as a stem cells source for more than 45% of APA patients and 60% of young adults, whereas children received mostly bone marrow and cord blood units. The observation of an increased incidence of chronic GVHD in APA patients has already been reported by Vignon et al. in a precedent study and is always an important matter due to the impact on quality of live (Vignon et al. 2017). As previously described, a high dose of Cyclophosphamide after HSCT reduced the risk of chronic GVHD, and also chronic extensive GVHD such as ATG did. Our results are consistent with previous studies on this point (Kröger et al. 2016; Martinez-Cibrian et al. 2020; Ruggeri et al. 2018; Luznik et al. 2012).

Moreover, we noted that allogeneic HSCT from 9/10 HLA matched unrelated donors resulted in a significantly worse OS than those from both 10/10 HLA matched unrelated donors and HLA identical sibling donors, which is mainly due to increasing NRM (Petersdorf et al. 2004; Flomenberg et al. 2004; Lee et al. 2009; Woolfrey et al. 2011; Horan et al. 2008). Cytomegalovirus serologic positivity for the recipient was also correlated to GRFS and grade III to IV acute GVHD, as previously described before the use of letermovir (Marty et al. 2017).

Donor age was higher in APA patients and young adults; besides, donor age was an independent factor in OS, EFS, NRM, GRFS and chronic extensive GVHD. According to previous publications, allogeneic HSCT from older donors could be associated with reduced OS (Kollman et al. 2001; Loren et al. 2006; Bastida et al. 2015; Ayuk et al. 2018; Shaw et al. 2018) for several reasons: on one hand, higher comorbidity and mobilization failure, on the other, to increased rates of acute and chronic GVHD, higher NRM and relapse rate (Kollman et al. 2001; Loren et al. 2006; Bastida et al. 2015). Recent study data from two works published in 2018 by Ayuk et al. and Shaw et al. showed the impact of the donor’s age and sex mismatch that could be comparable to a single HLA disparity (Ayuk et al. 2018; Shaw et al. 2018).

## Conclusions

Adolescent and post-adolescent patients, like young adults, have a greater risk of NRM and chronic GVHD than children after allogeneic HSCT for AML. They also have a higher cumulative incidence of relapse, even if age is not an independent factor of relapse. Therefore, this study suggests that APA patients with AML could be beneficially treated with a conditioning regimen based on myeloablative chemotherapy associated with bone marrow graft. Moreover, donor age and HLA compatibility should also be carefully assessed prior to the procedure. A future prospective comparative study is needed to confirm these results and to assess this important issue of conditioning and stem cell source choice in APA patients who received an allogeneic HSCT for AML.

## Supplementary Information

Below is the link to the electronic supplementary material.

Supplementary file1 (DOCX 14 kb)

## Data Availability

The dataset used and analyzed during the current study is available from the corresponding authors on reasonable request.

## Abbreviations

AML: Acute myeloid leukemia
ATG: Anti-thymoglobulin
APA: Adolescent and post-adolescent
BM: Bone marrow
CBF: Core binding factor
CI: Cumulative incidence
CIF: Cumulative incidence functions
CMV: Cytomegalovirus
CR: Complete remission
CR: Competitive Risk
DLI: Donor lymphocyte infusion
EFS: Event-free survival
GRFS: Graft-versus-host disease and relapse free survival
GVHD: Graft-versus-host disease
HSCT: Hematopoietic stem cell transplantation
MAC: Myeloablative conditioning
MRD: Minimal residual disease
NMDP: National marrow donor program
NRM: Non-relapse mortality
OS: Overall survival
PBSC: Peripheral blood stem cells
PT-Cy: Post-transplant high dose cyclophosphamide
RIC: Reduced intensity conditioning
SFGM-TC: Société francophone de greffe de moëlle et de thérapie cellulaire
SLE: Significance levels for entry
SLS: Significance levels for stay
TBI: Total body irradiation
TRM: Transplant-related mortality
