# Supplementary material for: Improved outcome in children compared to adolescents and young adults after allogeneic hematopoietic stem cell transplant for acute myeloid leukemia: a retrospective study from the Francophone Society of Bone Marrow Transplantation and Cell Therapy (SFGM-TC)
Source: J Cancer Res Clin Oncol. 2021 Sep 4;148(8):2083–97. doi: 10.1007/s00432-021-03761-w (PMC9293841; doi:10.1007/s00432-021-03761-w)
Supplement: Supplementary file 1 — Supplementary file1 (DOCX 14 kb) [file 432_2021_3761_MOESM1_ESM.docx]

**Supplementary Table 1: Causes of Deaths (except relapse)**

| **Causes of deaths (n, % of deaths)** | **Children**  **(n=196)** | **APA patients (n=289)** | **Young adults (n=647)** |
| --- | --- | --- | --- |
| Infections | **21 (10.7%)** | **53 (18.3%)** | **142 (21.9%)** |
| GvHD | **20 (10.2%)** | **40 (13.8%)** | **125 (19.3%)** |
| SOS | 5 (2.5%) | **14 (4.8%)** | **21 (3.2%)** |
| Pulmonary toxicity | **9 (4.6%)** | 8 (2.8%) | 17 (2.6%) |
| Rejection/Poor Graft Function | 1 (0.5%) | 3 (1%) | 12 (1.8%) |
| Haemorrhage | 6 (3%) | 13 (4.5%) | 20 (3.1%) |
| Cardiac toxicity | 3 (1.5%) | 3 (1%) | 9 (1.4%) |
| CNS toxicity | 2 (1%) | 7 (2.4%) | 12 (1.8%) |
| GI toxicity | 2 (1%) | 4 (1.4%) | 1 (0.1%) |
| Renal failure | 3 (1.5%) | 9 (3.1%) | 19 (2.9%) |
| Lymphoproliferative Disorder | 2 (1%) | 5 (1.7%) | 9 (1.4%) |
| « Multiorgan failure » | 15 (7.6%) | 30 (10.4%) | 79 (12.2%) |

APA: Adolescent and post-adolescent; CNS: Central Nervous System; GI: Gastro-Intestinal; GVHD: Graft Versus Host Disease; SOS: Sinusoïdal Obstruction Syndrome
